# Identification and management of mental health distress in Moroccan patients with cancer: Strategies adopted by oncology nurses and barriers to practice

**DOI:** 10.1002/cnr2.1985

**Published:** 2024-04-16

**Authors:** Amina Aquil, Mustapha Mouallif, Abdeljalil Elgot

**Affiliations:** ^1^ Laboratory of Health Sciences and Technology Higher Institute of Health Sciences, Hassan First University of Settat Settat Morocco

**Keywords:** identification, management, mental distress, oncology nurses

## Abstract

**Background:**

Adressing mental distress among cancer patients presents a substantial challenge in the delivery of oncology care.

**Aims:**

This present study aims to explore the nursing strategies for identifying and managing distress in cancer patients as well as the concomitant barriers that prevent them from achieving this task.

**Methods:**

This qualitative study is based on a semi‐structured interview with 25 practicing nurses in oncology.

**Results:**

Strategies used by nurses to identify mental distress in their patients include: receiving information, mobilizing interpersonal skills, and identifying causes of distress. When asked about the barriers that hinder the practice of identifying and responding to patients' distress, nurses reported facing several barriers that can be classified into three categories: health care system‐related barriers, patient‐related barriers, and nurse‐related barriers.

**Conclusion:**

Oncology nurses should benefit from specific training on the systematic assessment of mental distress in cancer patients, in order to improve the overall management of oncology patients.

## 1 INTRODUCTION

According to estimates from the International Agency for Research on Cancer, one in five people are at risk of developing cancer in their lifetime, and 1 in 8 men and 1 in 11 women die from it worldwide. Socio‐economic risk factors remain among the main factors behind this increase.^1^ According to the literature, mental health disorders are common in cancer patients and the psychological well‐being of this population remains one of the most explored issues in oncology. In addition, the psychological care of these patients has increasingly become seen as an important part of cancer care. Over the past 70 years, extensive research in oncology has shown that cancer has significant psychosocial consequences for patients and families in terms of psychiatric and psychosocial morbidity influencing the overall burden of the disease.^2^, ^3^

Some studies have shown that diagnosis and treatment of mental health disorders positively affects patients' adherence to cancer treatment as well as increases their survival rate.^4^, ^5^ Nevertheless, researchers have reported that many cancer patients do not benefit from detection and management of their mental health distress.^6^ Among the reasons for this problem is the nonrecognition of mental health disorders in patients by oncology health professionals.^7^ In this sense, a study, conducted among 102 cancer patients, found that the majority of them suffered from depressive symptoms, while less than a third of these patients had a detection of mental distress.^8^

Detection of distress is the essential step in assessment process for mental distress in oncology in order to ensure adequate management. Additionally, the role of the nurse, defined by the Canadian Association of Oncology Nurses, includes a thorough assessment of patient needs as well as facilitating continuity of care and decision‐making for patients, nurses showed considerable uptake of distress screening results.^9^ Considering that cancer patients frequently experience unaddressed and unsupported mental health‐related distress, and recognizing that oncology services in Morocco lack standardized screening and treatment protocols for mental health issues in cancer patients, this study is designed to investigate the strategies employed by Moroccan oncology nurses to identify and address distress in their patients, as well as the barriers they encounter in performing this role.

## 2 METHODS

### 2.1 Study design and participants

The present qualitative study was carried out over a period of 3 months (March 1, 2022 to June 1, 2022), at the level of four oncology centers in Morocco (Hematology and Oncology Center of the University Hospital of Marrakech, Oncology Department of the Ibn Rochd University Hospital Center Casablanca, Oncology Center of the Hassan II University Hospital (Fez) and Oncology Center Beni Mellal). Participants were invited by the head nurse of each oncology department, a total of 25 practicing oncology nurses were recruited and interviewed on the strategies they adopt to identify and manage mental health distress in cancer patients and the barriers that prevent them from practicing this task.

### 2.2 Procedure

Ethics approval was obtained from the Moroccan Association for Research and Ethics, Research Ethics Committee, (N° 06/REC/20) before the launch of the study. Then, a descriptive qualitative study was started in the oncology departments and spread over a period of 3 months. The interview, which lasted an hour on average, took place in an office within the oncology centers where each participant worked. First, the participants were informed about the study in question and they signed a consent form and accepted the audio recording of the interview. They then completed a socio‐demographic data form. Subsequently, a semi‐structured interview was used (Table 1), and the interviews were recorded and transcribed, with all identifiable information removed from the transcripts. Twenty‐five nurses participated in the study and this sample made it possible to obtain data saturation (17 nurses). When writing the manuscript, the authors respected the guidelines of consolidated criteria for reporting qualitative research (COREQ).^10^

**TABLE 1:** Interview schedule.

Axes of the interview	Questions
Screening for Mental Distress in Cancer Patients	Have you received training in psycho‐oncology or mental health? How many trainings and in what context?
	2 During your practice, do you assess the mental health of your patients? If yes, what strategies do you use for assessment?
	3 Within your department, is the assessment of mental health standardized or individualized based on patient categories such as pediatric, geriatric, or gender‐based?
	4 In your opinion, what are the key indicators for detecting and assessing the mental health of cancer patients?
	5 What are the barriers that prevent you from assessing mental disorders?
Management of Mental Distress in Cancer Patients	What strategies do you use to help the patient cope with this distress?
	2 What are the difficulties/barriers that hinder you from addressing the mental distress of cancer patients?”

### 2.3 Data analysis

Data collection and analysis took place simultaneously and coding of the transcripts was used. The analysis was inductive with codes and categories emerging from participants' stories and not preconceived codes. As the analysis continued, the descriptive codes were further distilled to capture the main themes and sub‐themes emerging from the nurses' narratives. Constant comparison was used to examine relationships within and between codes and categories, thoughts, reflections, and reactions throughout the process of collecting and analyzing data which was then used to inform the coding scheme. The research team held regular meetings to review emerging discoveries and maintain coherence in the evolving coding system throughout the data collection and analysis phases. Data collection stopped when the team determined that we were full and no new code had been created. Atlas ti 9 is the software we have been used to store and organize data This tool is qualitative data analysis (QDA) software that provides a platform for researchers to analyze and manage large volumes of text, graphics, audio, and video data. It is designed to facilitate the systematic analysis of unstructured data, including interviews, surveys, focus group transcripts, and other qualitative content. Indeed, it offers a range of tools for coding, organizing, and interpreting data, enabling researchers to uncover patterns, themes, and relationships within their datasets.^11^

## 3 RESULTS

### 3.1 Characteristics of the participants

The average age of the participants was 33.76 years with a 6.5‐year standard deviation (SD), the vast majority of nurses are female (60%), married (80%), and practicing nurses (84%) in different oncology units in Morocco. The average years of practice was 10.59 (6.52) and 4.35 (3.44) years of oncology practice. The majority of nurses have a professional nursing license (68%) and almost all do not have an oncology certification (80%) (Table 2).

**TABLE 2:** Sample characteristics (*n* = 25).

Characteristics	
Sex % (*N*)	
Man	40 (10)
Woman	60 (15)
Age: Average (standard deviation)	33.76 (6.5)
Marital status % (*N*)	
Married	80 (20)
Single	20 (5)
Oncology unit % (*N*)	
Hematology and Oncology Center of the University Hospital of Marrakech	32 (8)
Oncology Department of the Ibn Rochd University Hospital Center Casablanca	40 (10)
Oncology Center of the Hassan II University Hospital (Fez)	16 (4)
Oncology Center (Beni Mellal)	12 (3)
Current position % (*N*)	
Nurse practitioner	84 (21)
Head of Service	16 (4)
Years of practice: Average (standard deviation)	10.59 (6.52)
Years of practice in oncology: Average (standard deviation)	4.35 (3.44)
Last diploma obtained	
State diploma	20 (5)
Professional license	68 (17)
Master	12 (3)
Certification in oncology	
Yes	20 (5)
No	80 (20)

### 3.2 Strategies for identifying mental distress

When nurses were asked to discuss identifying distress in their oncology patients, they reported five main themes: (a) Get information directly from the patient; (b) Ask family members; (c) Communication and active listening; (d) Establish a trust relationship; (e) Look for the causes of distress.

**FIGURE 1:**
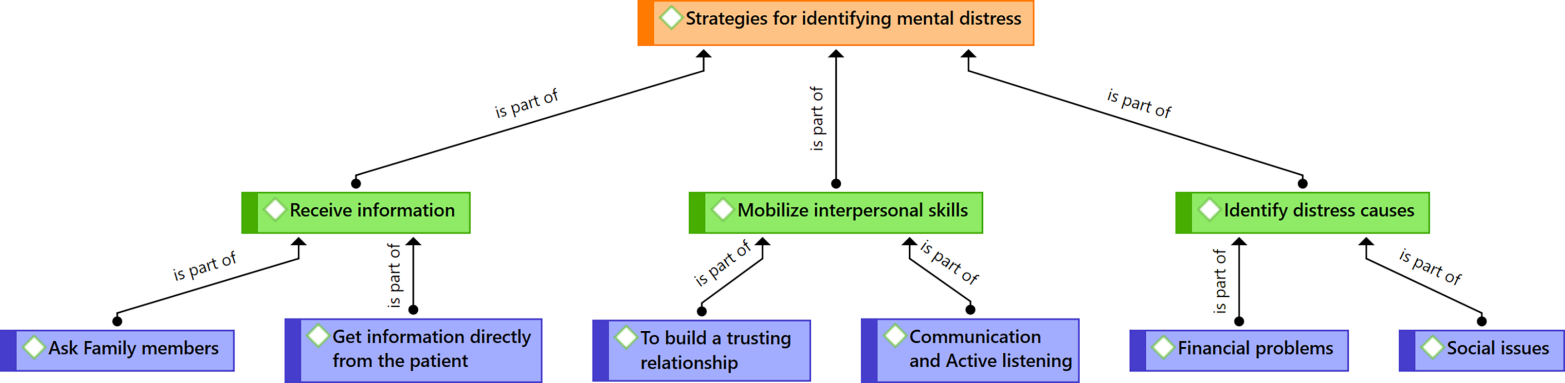
Strategies for identifying mental distress.

All nurses interviewed confirmed that there is no longer a standardized screening and assessment protocol for mental health distress in oncology departments. However, these participants reported adopting their own distress identification strategies. Each theme is described in more detail below with supporting quotes and presented in Figure 1:

#### 3.2.1 Get information directly from the patient

When nurses were asked about their strategies for identifying mental health distress in cancer patients, they reported a theme that involved requesting directly. Regarding this, a nurse said: “I am asking her direct questions: ‘How are you feeling today?’”. Another claimed in the same vein: “I ask them with questions. For example, I notice the woman is absorbed with a sad look I ask her,” “What is the matter? Why are you sad?”.

#### 3.2.2 Ask family members

Faced with difficulties in communicating with the patient, some nurses reported learning about the patient's mental health from family and friends. As one nurse mentioned: “When the patient seems reluctant to me, I ask his relatives about the symptoms of distress such as sleep disorders”.

#### 3.2.3 Communication and active listening

Some nurses reported that communication and active listening were part of their strategies for identifying mental health distress in patients. In this regard, a nurse explained: “I do not do an assessment based on standardized or validated tools but I do it in my own way… By talking to the patient, by analyzing the non‐verbal nature of patient.”

#### 3.2.4 Establish a trust relationship

Other nurses indicated that building trust was one of their strategies for assessing mental health distress in cancer patients. A nurse explained in this sense: “I always work on the bond of trust, the stronger this bond, the more I can know the patient's condition and his psychological suffering.”

#### 3.2.5 Look for the causes of distress

One nurse said she relied on a patient interview to research the sources and likely causes of patient distress. With this in mind, she reported: “The sources of the distress let me know if the patient has a tendency to be depressed… when the woman has just had her divorce letter after a hysterectomy… when a woman moves from a companion very far from the hospital and she does not have the means and leaves her children… here we are talking about the sources or the causes of mental distress.”

### 3.3 Strategies for managing mental distress

**FIGURE 2:**
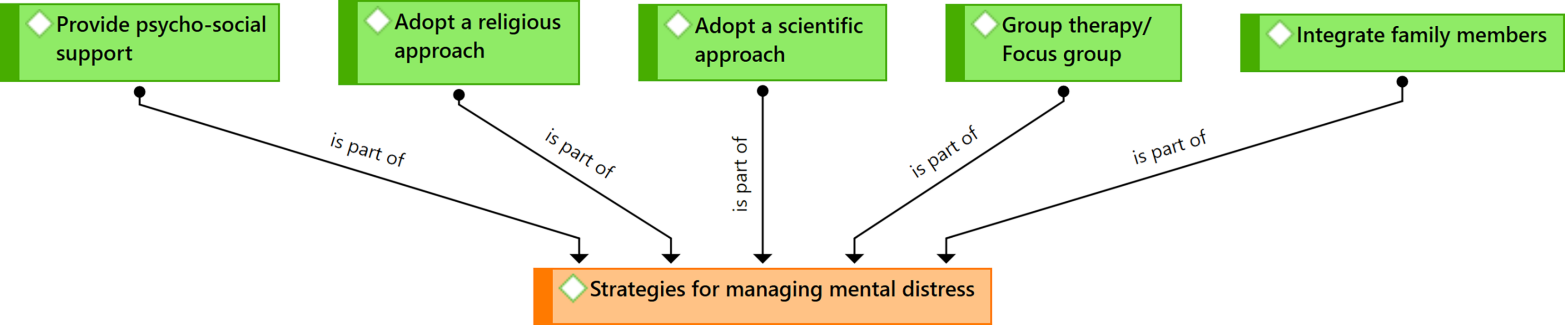
Strategies for managing mental distress.

Some participants reported that after identifying the distress, they used a number of strategies to deal with their patients' distress. The data coding allowed us to group the ratings into five themes: (a) Provide psycho‐social support; (b) Adopt a religious approach; (c) Adopt a scientific approach; (d) Group therapy/Focus group; (e) Integration of family members. The results are summarized in Figure 2 and each theme is explained in more detail below.

#### 3.3.1 Provide psycho‐social support

Nurses and social workers reported that one way they helped manage the patient's mental health distress was to try to build a supportive and compassionate relationship with their patients based on affective empathy, the act of soothing, relieving and reassuring the patient, giving him hope, encouraging him, strengthening him, and making him smile. Additionally, creating a climate of humor and trivializing the disease were also mentioned as strategies adopted by some participants.

Other participants indicated that they kept their kindness and supported the character of their patients. In this regard, a social worker explained: “As a strategy, I push the patient to express herself, and support her even if she speaks and tells things far from her therapeutic course, I listen to her, I accept her, don't block it, i avoid severe reactions.”

In the same vein of psycho‐social support, a head nurse said she was involved in a socio‐esthetic project allowing women with cancer to benefit from esthetic care in order to reduce their distress.

From another perspective, facilitating access to care was a way of contributing to the management of mental distress. In this regard, a nurse noted: “…I try to contribute to this care by facilitating the circuit for the patient because that also stresses him….”

#### 3.3.2 Adopt a religious approach

Participants noted that they used the religious approach to help patients cope with their distress. As one nurse remarked: “I always say an expression to my patients: ‘It is not the disease that kills the person’, I always refer to religion, I give examples of people who have died during an accident or people who die from strokes when there are people who had metastases and they healed anyway.”

#### 3.3.3 Adopt a scientific approach

In addition to the religious approach, nurses and social workers reported that they adopted a scientific approach to managing distress, mainly based on informing patients about the progress of science in terms of the treatment of distress disease and current survival rates. About this, a nurse said, “First the thing I do and I see it very important is information. When I explain to the patient that science has no limits and that cancerous disease has become like other chronic diseases it is curable and the treatment is effective it relieves her. Because the patient has information needs and emotional needs.”

#### 3.3.4 Focus group

Some nurses have indicated that they rely on group therapy to provide relief to distressed patients and give them hope. In this sense, a social worker explained: “Sometimes I use group therapy… For groups, I bring them together according to certain criteria, for example women in a group and men in a group. Sometimes I form groups according to age or people who have the same problem…. I take the example of a very positive person or a person who had an advanced stage but has healed, I ask him to speak to them and tell his story.”

#### 3.3.5 Integration of family members

Nurses said they were integrating the patient's relatives to address his broad‐spectrum distress. This integration is based on correcting rumors related to the disease, educating the family in its role of support and accompaniment, sex education and therapeutic education. As a head nurse explained during nurse consultation: “I bring the patients together after the first medical consultation, the patients come depressed, do not even know the trajectory of their care or their treatment. So, I gather the women in a room of 50 to 60 people, this number includes the patients and their companions such as the son or the husband of the wife, but I especially insist on the presence of the husband so that he understands and inform him on the intimate side… that his wife needs to be surrounded… that he be kinder and more attentive to her…. The woman too, must understand that she can continue her life and fight to be able to live. That there is progress in the therapeutic care… I give him feeding advice and correct bad rumors for the woman and for the family… I insist on the importance of the family support, it is necessary not to stigmatize the cancer woman. So it is important that the primary caregiver listens to us and understands either the son, the daughter or the partner to continue the normal life and understand that cancer is not a contagious disease and to continue the intimate relationship with the woman and c is very important for the woman to feel that she is supported ….”

### 3.4 Barriers to the assessment and management of mental distress

**FIGURE 3:**
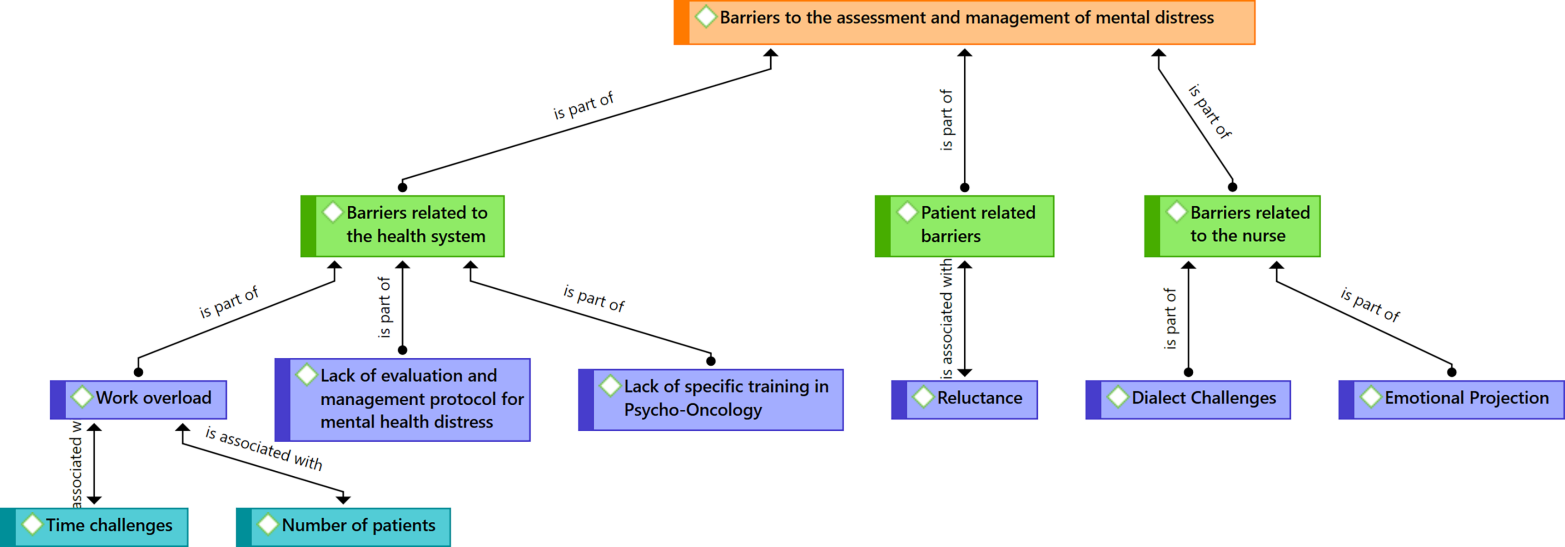
Barriers to the assessment and management of mental distress.

When the nurses were asked to discuss barriers to identifying distress and managing it, they stated three main themes: (a) Barriers related to the health system; (b) Barriers related to the patient; (c) Barriers related to the nurse (Figure 3).

#### 3.4.1 Barriers related to the health system

##### Lack of evaluation and management protocol for mental health distress

Some nurses said that the lack of tools to screen for distress, the absence of a protocol for the assessment and management of mental health problems in oncology services could be a barrier to developing. “carry out a systematic assessment of the distress as well as hamper its management.

##### Lack of specific training in psycho‐oncology

Oncology nurses indicated that they had not received any training in assessing mental health distress. In their view, the lack of knowledge and skill in psycho‐oncology was a significant barrier in helping their patients. Only two nurses who received continuing education in oncology care had the opportunity to have a psychology module during this training. Indeed, their knowledge on the subject was based on their personal experiences.

##### Work overload: Time challenges and the number of patients

Nurses reported that a major barrier to identifying mental health distress was a lack of time to sit down and listen to patients. Then faced with a huge number of patients with a shortage of human resources, they also reported being so overburdened with practical care responsibilities that they rarely had time to talk to patients about their psychological state.

#### 3.4.2 Patient‐related barriers

Reluctance: Asked about the barriers to dealing with mental distress, other nurses reported obstacles related to patients such as the refusal to share their suffering and the refusal of help offered by nurses or by the attending physician. As one nurse remarked: “When the patient is used to sharing her suffering, it allows me to talk to her and support her but when she is withdrawn and she no longer wants help it also blocks me… Sometimes I feel that the patient does not want to share her suffering and hides her vulnerability.”

#### 3.4.3 Barriers related to the nurse

##### Dialect challenges

In connection with the challenge identified above, nurses reported that the dialect was a barrier to identifying and managing distress. On this subject, a nurse claimed: “For example, I work in Tangier, I welcome many patients of ‘Rifi’ origin, I do not speak the dialect of their region, I already find it difficult to approach subjects with patients… I only speak with relatives who understand the Arabic dialect… the latter constitutes a barrier, so I cannot discuss with these kind of patients the state of their mental health, their feelings… especially since in this case the patient remains silent and his loved one answers in his place so I cannot know the patient's anxieties.”

##### Emotional projection

Other nurses have reported that their emotional projection of patients may act as a barrier to assessing mental health distress in these patients. On this problem, a nurse remarked: “… I do the projection in each patient I see a mom, I see a sister, a dad… Sometimes it blocks me, I avoid feeling what they have. if not, I will cry with them….”

Faced with these barriers as well as the absence of psychiatrists and psychologists within certain oncology services, the nurses expressed their wish to have favorable conditions in order to be able to carry out a systematic assessment of distress linked to mental health with their cancer patients.

## 4 DISCUSSION

Mental distress, such as anxiety and depression, is a common issue in cancer patients.^12^ Several studies have demonstrated that psychosocial distress ranks among the primary concerns for individuals facing cancer, leading to difficulties in adhering to treatment and causing them to withdraw from family and social support systems.^13^, ^14^ Although it has been recommended that nurses take on the role of assessing distress due to their privileged position within oncology services compared to other health professionals, as well as the positive impact of this task on nursing practice, little is known about how nurses assess distress, respond to patients' needs, and the challenges they encounter in their practice.^15^, ^16^

The primary objectives of the present study were to investigate how oncology nurses identify distress in their patients, examine the strategies they employ to respond to their patients' distress, and explore the challenges they face when addressing the mental distress needs of their patients. Nurses employ various strategies to identify mental distress in their patients, which encompass information gathering, utilization of interpersonal skills, and identifying the underlying causes of distress. In responding to patients' needs, nurses reported providing psychosocial support, incorporating religious and scientific approaches, conducting focus groups, and involving the patient's family. Nurses also identified three categories of barriers that impede the identification and response to their patients' distress. These barriers encompass issues related to the healthcare system, such as the absence of standardized assessment and distress treatment protocols in oncology, the high workload due to the number of patients and time constraints, and the lack of specific training in psycho‐oncology. Additionally, barriers stemming from patient‐related factors include patient reluctance, while barriers linked to the nurses themselves involve emotional projection and challenges in communication.

In the same perspective of our results, a study by Granek et al indicated that nurses in oncology departments relied on questioning patient to assess distress, while the lack of training in mental health and time constraints are the main obstacles to this assessment.^17^ Patient reluctance to ask the nurse's questions has been mentioned as a barrier to helping patients, other studies have reported that the use of random assessment strategies can lead to misdiagnoses.^18^ In this sense, a study showed that nurses trained in interpersonal skills were better able to assess distress in cancer patients than those who were not trained.^19^

Overall, the nurses in our study were not trained to screen for and respond to mental distress in patients and they confirmed that there was no protocol for assessing and managing the distress within their departments. Nonetheless, they reported the frequency of distress in their patients and mentioned the strategies they adopt to identify and cope with it. These data are encouraging insofar as nurses, despite the obstacles, have recognized the importance of managing distress and have sought solutions to alleviate it. However, these results remain worrying, because the evaluation remains non‐standardized and not systematic, each nurse is based on his own knowledge and his own skills to know how to assess the distress and how to meet the patient's needs.^20^

As previously discussed, it is imperative to conduct mental distress screening for cancer patients as an integral component of their comprehensive care.^21^ Screening enables the referral of patients to psycho‐oncology specialists, ensuring the effective management of cancer‐related emotional challenges. Beyond its potential to enhance patient well‐being and quality of life, there is substantial evidence suggesting that unaddressed distress in patients can result in adverse outcomes, such as non‐compliance with treatment regimens,^22^ compromised anti‐cancer care,^23^ reduced satisfaction with healthcare,^24^ prolonged hospital stays,^25^ increased healthcare visits,^26^ and elevated cancer‐related mortality.^4^ Consequently, mental distress screening carries not only personal but also economic and systemic implications, profoundly impacting patients and their healthcare providers. To promote the routine adoption of evidence‐based mental health screening, it is imperative to educate stakeholders about the significance and rationale underlying the identification of distress.^20^

To enhance the accuracy of mental health disorder diagnosis, clinical approaches should encompass the systematic utilization of reliable tools tailored to the Moroccan context for distress screening. These tools may include the Hospital Anxiety and Depression Scale (HADS),^27^ a 14‐item self‐report questionnaire assessing anxiety and depression symptoms experienced by patients in the past week. A HADS total score exceeding 15 has demonstrated good sensitivity and specificity for identifying anxiety and depressive disorders in cancer patients.^28^

Another widely employed tool is the Distress Thermometer,^29^ a straightforward self‐report measure represented as a scale ranging from 0 to 10, anchored at 0 with “No Distress” and at 10 with “Extreme Distress.” Patients are asked to indicate their level of distress on this scale for the past week. A score of 4 or higher signifies the need for intervention. This measure has been extensively employed in cancer patient research and is recommended for clinical use.^30^ Patients can self‐administer this screening, optimizing the utilization of limited treatment time. Only patients scoring above the cutoff should be flagged, enabling healthcare professionals to engage in focused discussions with them.

### 4.1 Study limitations

Our study has certain limitations. While our in‐depth qualitative methodology provided valuable insights into the identification and management of mental distress in cancer patients by oncology nurses, it did not allow us to determine the actual frequency of clinical distress assessments or the number of patients referred for psychosocial care. Despite our efforts to gather this information, no objective data in the form of health records were available to ascertain the exact number of patients who received psychosocial care.

### 4.2 Clinical implications

Systematic assessment of mental distress in oncology may have several benefits and improve clinical implications for oncology nurses. First, the systematic assessment of mental distress allows early identification of patients who may have mental health needs. This allows oncology nurses to quickly identify patients who may require further assessment and psychosocial intervention. Early detection can help prevent a deterioration in the patient's mental state and allow earlier and more effective intervention. Then, the individualization of care, by systematically evaluating the mental distress of cancer patients, nurses can better understand their needs and adapt care accordingly. The assessment of mental distress takes into account the psychosocial, emotional, and cognitive factors that can influence the overall health of the patient. This allows for the provision of individualized care that meets the specific needs of the patient, including appropriate psychosocial and supportive interventions. Then, the systematic assessment of mental distress helps to recognize and treat psychological symptoms such as anxiety, depression, fear, or hopelessness in cancer patients. Oncology nurses may work collaboratively with a multidisciplinary care team to implement distress management strategies, such as psychological support interventions, referrals to mental health specialists, or stress management programs. This helps to improve the psychological well‐being of patients and promote a better quality of life. Additionally, systematic assessment of mental distress can facilitate communication between oncology nurses and patients. By asking questions about mental distress, nurses open the door to deeper discussions about patients' emotions, concerns, and needs. This can strengthen the therapeutic relationship, foster empathy and trust, and enable nurses to provide emotional support and attentive listening to patients. Also, this systematic evaluation of mental distress makes it possible to measure the results of the psychosocial interventions implemented for patients. Nurses can regularly assess patients' mental distress to monitor the effectiveness of interventions and adjust care accordingly. This allows patients' progress in their mental health to be tracked and necessary changes to be made to optimize their well‐being. The systematic assessment of mental distress in oncology enables oncology nurses to detect mental health needs early, individualize care, manage psychological symptoms, improve communication with patients, and follow up the effectiveness of interventions. This contributes to improved clinical implications by providing holistic support to cancer patients.

## 5 CONCLUSIONS

In conclusion, this study underscores the crucial importance of early identification of mental distress in cancer patients. While current strategies employed by oncology nurses, such as gathering information and utilizing interpersonal skills, show promise, additional efforts are required. Targeted training, aimed at enhancing communication skills and recognizing treatable disorders like anxiety and depression, is indispensable. Furthermore, proactively directing at‐risk patients to specialized mental health care, including psychotherapy and psychopharmacology, can narrow treatment gaps. Investing in the ongoing education of oncology nurses and promoting referrals to specialized care will significantly contribute to enhancing the overall care of oncology patients, thereby addressing current gaps in the provision of psychosocial support.

## AUTHOR CONTRIBUTIONS

**Amina Aquil:** Conceptualization (lead); data curation (lead); formal analysis (lead); investigation (lead); methodology (lead); writing – original draft (lead); writing – review and editing (lead). **Mustapha Mouallif:** Methodology (equal); supervision (supporting); writing – review and editing (supporting). **Abdeljalil Elgot:** Formal analysis (equal); methodology (supporting); supervision (lead); validation (lead); writing – review and editing (equal).

## CONFLICT OF INTEREST STATEMENT

The authors have stated explicitly that there are no conflicts of interest in connection with this article.

## ETHICS STATEMENT

Ethical approval was obtained from the Moroccan Association for research and ethics, Research Ethics Committee, (N° 06/REC/20). All procedures performed in this study involving human participants were in accordance with the ethical standards of the institutional and national research committee. Confidentiality and anonymity criteria were met as charted by the declaration of Helsinki and its later amendments.

## Data Availability

Data sharing is not applicable to this article as no new data were created or analyzed in this study.
